# Medical cannabis for the treatment of fibromyalgia syndrome: a retrospective, open-label case series

**DOI:** 10.1186/s42238-021-00060-6

**Published:** 2021-02-17

**Authors:** Manuela Mazza

**Affiliations:** Department of Anaesthesiology, Critical Care Medicine and Pain Medicine, Nuovo Ospedale degli Infermi, Via dei Ponderanesi 2, Biella, Ponderano Italy

**Keywords:** Medical cannabis, Herbal cannabis, Fibromyalgia treatment, Musculoskeletal pain, Open-label study, Case series

## Abstract

**Background:**

The use of cannabis for treating fibromyalgia syndrome (FMS) has not been comprehensively investigated. Thus, we have assessed the efficacy and adverse events (AEs) of short- and long-term medical cannabis (MC) treatment for FMS.

**Methods:**

Data were obtained from medical reports archived in the pain clinic of Ponderano (Italy; retrospective study). FMS patients, who were resistant to conventional therapy, received licensed MC with various Δ-9-tetrahydrocannabinol (THC) and cannabidiol (CBD) content, as powdered whole flowers (decoction or vaporization) or oil extracts. Demographic and clinical parameters, including Numerical Rating Scale (NRS), Oswestry Disability Index (ODI), Hospital Anxiety and Depression Scale, Widespread Pain Index (WPI), Severity Score (SyS), and side effects, were obtained after 1, 3, and 12 months. Data were analyzed with Wilcoxon signed-rank tests for paired data.

**Results:**

Thirty-eight patients were included. Thirty, 18, and 12 patients continued therapy for 1, 3, and 12 months, respectively. Significant improvements (*p* < 0.01) were observed in NRS, ODI, WPI, and SyS at 1 month; in NRS, ODI, and WPI at 3 months; and in NRS, ODI, and SyS at 12 months. Therapy was interrupted by 17 patients (48.6%) owing to nonserious AEs according to the FDA. The most common side effects were mental confusion (37%), dizziness (14%), nausea/vomiting (14%), and restlessness/irritation (14%). The median daily dose of milled flowers administered as THC-dominant MC and hybrid MC (with similar THC/CBD ratio) was 200 mg/day and 400 mg/day, respectively. After 3 months of titration, the median content of THC administered with THC-dominant MC cultivars was 46.2 mg, and of THC + CBD administered as a hybrid MC cultivar, was 23.6 mg + 38 mg. At 3 months, median THC content administered in the oil extract of the THC-dominant MC cultivars was 9.7 mg, while that of THC + CBD administered in the oil extract of the hybrid MC cultivars was 1.8 mg + 2 mg.

**Conclusions:**

MC may represent an alternative treatment for patients with FMS who are unresponsive to conventional therapy. However, its application may be limited by the incidence of nonserious AEs.

## Background

Fibromyalgia syndrome (FMS) is a common chronic pain syndrome that significantly impacts patient quality-of-life. FMS is characterized by widespread musculoskeletal pain, sleep and mood disorders, fatigue, cognitive disorders, and various somatic symptoms (Bellato et al. 2012); however, it is not associated with signs of tissue inflammation, deformity, or damage. Normal laboratory and medical tests are often suggestive of FMS; however, its pathogenesis has not yet been clearly identified (Schmidt-Wilcke and Clauw 2011). The prevalence of FMS in the general population varies from 0.5 to 7% (Croft 2002; Vincent et al. 2013) with a female-to-male ratio of 3:1 (Queiroz 2013; Wolfe et al. 1995). FMS typically occurs between 40 and 60 years of age (Branco et al. 2010; Mc Beth et al. 2001), while the genetic etiology of FMS remains unclear (Arnold et al. 2013).

The diagnostic criteria for FMS were revised by the American College of Rheumatology (ACR) in 2010 (Wolfe et al. 2010) and subsequently modified in 2011 (Wolfe et al. 2011) and 2016 (Wolfe et al. 2016), in which palpation of the 18 tender points was removed as a requirement for FMS diagnosis. Meanwhile, the polysymptomatic distress score (PDS) (Wolfe et al. 2013; Wolfe et al. 2015; Yaseen et al. 2017; Wolfe et al. 2018), which combines the scores from Widespread Pain Index (WPI) and the Symptom Severity Score (SSS), is included as diagnostic criteria.

Currently, the recommended drugs for FMS treatment include antidepressants, anticonvulsants, weak opioids, and muscle relaxants (Macfarlane et al. 2016); however, many patients exhibit poor responses to therapy and various side effects (mental confusion, vertigo, dry mouth, constipation, and increased blood pressure), affecting patient compliance (Macfarlane et al. 2016). Although the use of amitriptyline, a tricyclic antidepressant, is associated with a 30% reduction in pain intensity, its effect on sleep disturbance is poor (Macfarlane et al. 2016). Similar effects were observed in other studies investigating the effect of anticonvulsants and serotonin-noradrenalin reuptake inhibitors on symptom relief (Üçeyler et al. 2013; Lunn et al. 2014).

Medical cannabis (MC) is derived from *Cannabis sativa* L. The flowers of *C. sativa* L. contain over 500 chemical components, with more than 100 types of phytocannabinoids (Mechoulam et al. 1976; Grotenhermen and Russo 2002).

Many reviews and meta-analyses have been published on the use of MC by patients with chronic pain (Aviram and Samuelly-Leichtag 2017). The most encouraging results have involved the use of MC in neuropathic pain (Andreae et al. 2015). In a recent study, treatment of chronic pain with MC resulted in improvements in pain and functional outcomes and a significant reduction in opioid use (Haroutounian et al. 2016). Another study suggested the potential use of low-dose medical marijuana with traditional analgesics for refractory neuropathic pain (Deshpande et al. 2015). However, the trials were limited owing to their short duration, variability in dosing, and lack of functional outcomes (Deshpande et al. 2015). Moreover, a previous systematic review described the benefits of cannabis-based medicines for neuropathic pain, although the benefits must be weighed against the disadvantages (Banerjee and McCormack 2019). Meanwhile, a recent Cochrane review indicated the absence of high-quality evidence related to the efficacy of cannabis-based products, including herbal cannabis (marijuana), for patients with chronic neuropathic pain (Mücke et al. 2018). Additionally, certain adverse events (AEs), particularly somnolence or sedation, confusion, and psychosis, may limit the clinical application of cannabis-based medicines (Mücke et al. 2018). A recent prospective observational study of 367 patients with FMS who were administered MC (oil extract, flower capsules, or cigarettes) and followed-up for 6 months, reported a reduction in pain intensity with mild side effects, including dizziness, dry mouth, and gastrointestinal symptoms. The median cannabis dosage was initially at 670 mg/day and 1000 mg/day at 6 months (Sagy et al. 2019).

Recently, cannabis has been approved for medical use in specific states in the USA, as well as in certain European countries (Troutt and DiDonato 2015; Ko et al. 2016). For instance, in Italy, MC is licensed by the National Cannabis Agency of the Ministry of Health (REGIONE PIEMONTE BU8S1 2016). Specifically, in the Piedmont region of Italy, with implementing decrees, various clinical conditions unresponsive to conventional treatments have been included among those with indication to therapy with MC borne by the National Health System (NHS). One of these clinical conditions is chronic pain unresponsive to nonsteroidal anti-inflammatory drugs, steroids, or opioids. Other clinical conditions approved for the use of MC borne by NHS are diseases associated with spasticity (e.g., multiple sclerosis and spinal cord injury), nausea and vomiting induced by chemotherapy, radiotherapy and HIV, to increase appetite in patients affected by cancer, acquired immunodeficiency syndrome, and anorexia, and alleviate hypertension in patients with glaucoma and to treat tic in Gilles de la Tourette syndrome. Hence, MC treatment in the Piedmont region is facilitated by the National Health Service (NHS), which is prescribed by the attending physician (Decreto ministeriale 23 gennaio 2013).

To date, few studies have described the use of cannabis in patients with FMS (Fiz et al. 2011; Piper et al. 2017) and the available studies present data for patients administered unlicensed cannabis. Thus, information regarding the type and dosage of cannabis used is lacking (Fiz et al. 2011; Piper et al. 2017; National Pain Report 2014). Moreover, although recent randomized clinical trials have investigated the use of synthetic cannabis (Skrabek et al. 2008; Ware et al. 2010) or herbal cannabis, only short-term effects were evaluated (van de Donk et al. 2019); similarly, a recent review exclusively analyzed trials evaluating short-term effects (Walitt et al. 2016). Another recent study, examining the effects of MC in 30 Israeli FMS patients (Habib and Artul 2018) reported that MC elicited favorable effects with few AEs (Habib and Artul 2018). However, no such studies have been conducted in Italian FMS patients.

Thus, herein, data from Italian FMS patients taking MC were retrospectively evaluated to assess the effects of licensed MC on pain intensity, disability, widespread pain, disease severity, and mood disorders. The potential AEs of cannabis were assessed after short- (1 and 3 months) and long- (12 months) term treatment, and the median dosage per day of cannabis, as well as the THC and THC + CBD content required to reduce pain, were determined. The present study provides a new insight and novel data regarding MC treatment specific for FMS patients.

## Methods

### Study design

This was a retrospective study examining the analgesic efficacy of MC and AEs in adult Italian patients diagnosed with FMS who were deemed resistant to conventional drugs (e.g., tramadol, amitriptyline, duloxetine, and pregabalin).

### Patients and eligibility

This study was carried out at the pain clinic of Nuovo Ospedale degli Infermi of Ponderano (Biella, Italy). Data from patient files were assessed between June 1, 2016, and October 31, 2018. During this period, 75 patients presented at the pain clinic with a previous FMS diagnosis. MC therapy was offered to patients who had previously used conventional FMS drugs. Patients who provided written informed consent to begin MC treatment were asked to follow the clinical practice schedule comprising repeated prescription of cannabis once per month. A health report was generated for each patient once per month and archived as MC was recently approved as a therapeutic option in Italy.

After the local ethics committee’s approval (CE 168/18) and patient’s informed consent, clinical information collected throughout the normal course of clinical care by the attending clinician (the referee for MC therapy of the hospital) at visits conducted after 1, 3, and 12 months of MC therapy, were stored using Microsoft Excel.

Eligible patients included those visiting the pain clinic and meeting the following inclusion criteria: written informed consent; age >18 years; diagnosis of FMS according to the ACR diagnostic criteria of 2010 and validated by a rheumatologist, including a WPI ≥ 7 (scale, 0–19) and an SyS ≥ 5 (scale 0–12) or a WPI of 3–6 with an SyS ≥ 9; symptoms present at a similar level for at least 3 months with no disorders to otherwise explain the pain; resistant or intolerant to the following conventional pharmacological treatments (Macfarlane et al. 2016):

- Amitriptyline usually 25 mg/day (EULAR recommendation: weak for at low-dose, 100% agreement)
- Duloxetine 60 mg/day (EULAR recommendation: weak for, 100% agreement)
- Pregabalin at least 75 mg twice a day to 150 mg twice a day (EULAR recommendation: weak for, 94% agreement)
- Gabapentin at least 300 mg thrice a day (EULAR recommendation: research only, 100% agreement)
- Tramadol at least 100–150 mg/day (EULAR recommendation: weak for, 100% agreement)
- Acetaminophen 1,000 mg thrice a day, used clinically even with little evidence of efficacy

Resistant or intolerant patients were those who developed AEs to conventional pharmacological drugs or continued to experience moderate-severe pain scores according to numerical rating scale (NRS ≥ 4) after at least 3 months of conventional pharmacological therapy.

The exclusion criteria were as follows: severe cardiopulmonary disease, severe liver disease or chronic hepatitis C, severe renal impairment, history of drug or alcohol addiction, psychiatric disorders or family history of schizophrenia, and women planning pregnancy or those who were pregnant or breast-feeding (no urine/blood pregnancy tests were performed before the initiation of MC treatment).

### Treatment regimen

MC was administered as an adjunct therapy, and the baseline therapy was gradually decreased or interrupted as patients reported alleviation of their pain. The physician decided on the prescribed MC type and dose of herbal MC in milligrams. Accordingly, a one-month supply of MC was prepared by the chemist. The starting dose of the milled flowers in the sachet was 50 or 100 mg twice per day according to the patient’s sensitivity to drugs (patients who previously developed adverse side effects to drugs started at a dose of 50 mg). The dose could then be increased by the physician as needed to approximately 50–100 mg (100 or 200 mg/day). In the case of cannabis olive oil extract, patients were instructed to gradually increase their dosage at small intervals, i.e., a single oil drop every 3–4 days until the therapeutic effect was attained. To avoid AEs, patients were instructed to use the dosage that did not cause side effects. The titration period lasted from 1 to 3 months.

At the beginning of the study, the type of cannabis prescribed by the physician depended on the availability of the MC cultivar at the hospital pharmacy. When both types of cannabis, i.e., THC-dominant cultivar and hybrid MC cultivar (with a similar THC/CBD ratio), were available, the first choice was the hybrid MC cultivar to reduce the risk of side effects due to THC. The patients consumed only one cultivar at a time (during the 1-month period), however, could take both types of MC cultivars using different routes of administration over the course of the treatment.

### Ethical considerations and source of cannabis

The study was approved by the local ethics committee of Eastern Piedmont (CE 168/18). All eligible patients provided informed consent after the study was explained by a physician. All eligible patients opted to participate in the study and signed a consent form. Patients were not compensated for participation in this study. The cannabis was provided by the hospital pharmacy at the institution where the study was conducted. Five varieties of MC were used in the study: FM1, FM2 (Military Chemical and Pharmaceutical Institution of Florence, Italy); Bediol, Bedrocan (Bedrocan International BV, Veendam, the Netherlands); and Pedanios (AURORA Cannabis Enterprises Inc., Canada; Table 1). MC was provided by the NHS, as prescribed by national and regional laws.

**Table 1:** Medical cannabis cultivars prescribed to 38 patients with fibromyalgia syndrome at an Italian pain clinic

Cannabis	Origin	THC content (%)	CBD content (%)
**FM2**	Military Chemical and Pharmaceutical Institution of Florence, Italy	5–8	7.5–12
**Bediol**	Bedrocan International BV (Veendam, the Netherlands)	6	8
**FM1**	Military Chemical and Pharmaceutical Institution of Florence, Italy	13–20	< 1
**Bedrocan**	Bedrocan International BV (Veendam, the Netherlands)	22	< 1
**Pedanios**	AURORA Cannabis Enterprises Inc. (Canada)	17–26	< 1

*MC* medical cannabis, *THC* Δ-9-tetrahydrocannabinol, *CBD* cannabidiol

MC comprised dried, milled homogenized flowers or whole flowers of *C. sativa*. All plants were cultivated under standardized conditions according to the requirements of good manufacturing practices.

### Preparation and administration of cannabis

FM2 and FM1 were delivered to the hospital pharmacy as minced flowers. Thereafter, the doses of cannabis prescribed by the physician were prepared as sachets by the chemists. Bedrocan, Bediol, and Pedanios were delivered as whole flowers and finely minced for decoction (administered orally) or roughly minced for vaporization by the chemist. Oil was purchased from an outside private pharmacy (Farmacia Ternelli, Reggio Emilia) where it was prepared by the Romano-Hazekamp technique using funds provided by the hospital. The oil extract was supplied to the patient by the hospital pharmacy and was paid for by NHS. All MC products were accompanied by an analysis form upon purchase.

All cannabinoids in the plant were primarily present in their acidic form. Application of heat was required for the decarboxylation of the cannabinoid acids into their active forms (THC acid into THC; CBD acid into CBD) (ElSohly and Gul 2014).

For administration, the decoction was administered orally after preparation by boiling the contents of a sachet containing minced MC in 200 mL water and 30 mL of milk for 15–20 min (Hazekamp et al. 2007). Alternatively, the cannabis was administered by vaporization using a cannabis vaporizer at a temperature of 210°C (Gieringer et al. 2008). Finally, sublingual administration was performed using olive oil extract prepared by the Romano-Hazekamp technique (Romano and Hazekamp 2013).

### Data collection and endpoints

After obtaining consent, the demographic and clinical parameters of patients were extracted from medical reports and store using Microsoft Excel. Demographic data included gender, age, time from diagnosis of FMS and prescription of MC, body mass index (BMI), and comorbidities. The primary endpoint, pain relief, was evaluated with an NRS (a unidimensional measure of pain intensity). NRS is an 11-point scale used by patients to report pain and is designed for adults and children aged > 10 years old, with 0 indicating no pain and 10 indicating maximum pain, as follows: 0, no pain; 1–3, mild pain (nagging, annoying, interfering slightly with daily life activities); 4–6, moderate pain (significantly interfering with daily life activities); and 7–10, severe pain (disabling, unable to perform daily life activities) (Haefeli and Elfering 2006). The patients were asked to rate the average intensity of pain over the past week. Analgesic effects were considered when there was a reduction in pain intensity by at least 30%.

The secondary endpoint, AEs, was evaluated by the physician during monthly follow-up visits. The physician investigated whether the AEs reported by the patients were directly induced by MC or were already present. This assessment was used to determine whether treatment with MC should be continued.

Other secondary endpoints included disability, mood disorders, and severity of FMS. Disability was evaluated using a validated Italian version (Monticone et al. 2009) of the Oswestry Disability Index (ODI), a test evaluating disability in patients according to intensity of pain and its impact on daily activities (personal care, weight-lifting, walking, sitting, standing, sleeping, sexual activities, socialization, and traveling). Scores for disability were as follows: minimal disability, 0–20%; moderate disability, 21–40%; severe disability, 41–60%; crippling disability, 61–80% (pain impinges on all aspects of life); and complete disability, 81–100% (patients are either bed-bound or exaggerating their symptoms). Furthermore, ODI was applied in place of the Fibromyalgia Index Questionnaire as most patients visiting the pain clinic had lower back pain. ODI has not been previously used in patients with FMS, except for a recent study evaluating the prevalence of the FMS phenotype in patients with spinal pain (Brummett et al. 2013). Mood disorders were evaluated with the Hospital Anxiety and Depression Scale (HADS), a 14-item measure designed to assess anxiety and depression symptoms. The test identifies patients with anxiety and depression and stratifies them into four categories: 0–7, normal; 8–10, mild; 11–14, moderate; and 15–21, severe; the version used in this study had been previously validated in Italian patients (Costantini et al. 1999), as well as for hospitalized patients and mood disorders in outpatients (Habib and Avisar 2018). The severity of FMS was evaluated using the WPI, which assesses pain regions with a score between 0 and 19, and the SyS score, which assesses fatigue, waking unrefreshed, cognitive symptoms, and other somatic symptoms (scored between 0 and 12) (Wolfe et al. 2016; Wolfe et al. 2013; Wolfe et al. 2015; Yaseen et al. 2017; Wolfe et al. 2018).

Other clinical data collected included the dosage of cannabis reducing pain by at least 30%, route of administration, and development of tolerance (the need to increase dose after reaching analgesic dosage). Interruption of conventional FMS drugs during MC treatment was also evaluated. Complete drug interruption was considered significant. The use of the drug as everyday therapy was considered drug use, whereas rescue therapy was defined as occasional use of the drug. Evaluation of signs of withdrawal (Habib and Avisar 2018), such as loss of appetite, irritability, insomnia, or anxiety, was also performed to determine the possibility of addiction in patients who continued MC for at least one month and stopped treatment for any reason.

The validated Italian versions of ODI and HADS questionnaires were self-administered by the patient before medical examinations. The WPI or SyS was self-administered by the patient. NRS, side effects, other analgesic drug use, and signs of withdrawal were examined by the physician at each monthly follow-up.

### Statistical analysis

Descriptive statistics of the clinical parameters were assessed before and during MC treatment. Additionally, the dosages of milled MC, THC, and THC + CBD in the sachets and oil drops were measured. All demographic data were reported as median and interquartile range (IQR) and mean and standard deviation (SD).

For statistical analysis, Wilcoxon signed-rank tests for paired data were used to compare the parameters (NRS, ODI, HADS, WPI, and SyS) before cannabis therapy and after 1, 3, and 12 months of MC therapy. The data are from per protocol analyses. Results with *P* values < 0.01 were considered significant. No calculation of required sample size was done because it was a post hoc study. IBM SPSS software, version 25.0, was used for the statistical analysis.

## Results

### Patient characteristics

Seventy-five patients (72 women and three men) diagnosed with FMS visited the pain clinic during the study period. The median age was 55 years. The median time from diagnosis to consultation at the pain clinic was 66 months. Among these 75 patients, 38 (36 women and two men) were deemed eligible to participate in the current study. The median age was 56 years (range: 31–74 years); 3% of patients were < 40 years old, 71% were 40–65 years old, and 26% were > 65 years old. The excluded patients had a median age of 54 years, and median time between diagnosis of FMS and consultation at the pain clinic of 36 months. Most excluded patients did not meet all inclusion criteria or had not tried all conventional FMS drugs.

The demographic and clinical parameters (Table 2), comorbidities (Table 3), and associated pain syndromes (Table 4) of the 38 patients included in the study were evaluated. The most frequent comorbidities were hypovitaminosis D, with or without osteoporosis/osteopenia (44.7%), hypertension (39.5%), and depression (31.6%). The most frequently associated pain syndromes were lower back pain (23.7%), cervical pain (21.1%), and headache (21.1%).

**Table 2:** Demographic and clinical characteristics in 38 Italian patients with fibromyalgia syndrome treated with medical cannabis

Parameter	***N*** = 38			
Gender, female: male	36:2			
	**Mean**	**± SD**	**Median**	**IQR**
Age, years	56.6	9.8	56	10.75
Months between diagnosis of FMS and prescription of MC	91.1	73.5	78	84
Body mass index (Kg/m^2^)	25.8	5.8	24	5.5
Numerical Rating Scale	8.2	1.4	8	1.5
Oswestry disability index	55.5	17.6	55	29.5
HADS anxiety	12	4.5	11.5	8
HADS depression	11.7	4.6	12.5	7.25
Widespread Pain Index	14.4	4.1	15	7
SyS	10.1	1.4	10.5	1.5

*N* number of patients, *±* plus/minus, *SD* standard deviation, *IQR* interquartile range, *FMS* fibromyalgia syndrome, *MC* medical cannabis, *HADS* Hospital Anxiety and Depression Scale, *Sys* severity score

**Table 3:** Associated comorbidities in Italian fibromyalgia patients treated with medical cannabis

Comorbidities	***N*** = 38	
	Patients	%
Hypovitaminosis D with or without osteoporosis/osteopenia	17	44.7
Hypertension	15	39.5
Depression	12	31.6
Osteoarthritis	11	28.9
Hiatal hernia	8	21.1
Hypothyroidism	6	15.8
Hypercholesterolemia	6	15.8
Tachycardia/atrial fibrillation	4	10.5
Irritable colon	4	10.5
Diabetes	4	10.5
Hyperuricemia	4	10.5
Psoriasis	3	7.9
Carpal tunnel syndrome	3	7.9
Raynaud syndrome	3	7.9
Pulmonary thromboembolism	2	5.3
Liver disease	2	5.3
Glaucoma	2	5.3
Endometriosis	2	5.3
Kidney failure	1	2.6
Ischemic heart disease	1	2.6
Skin fungus	1	2.6
Vasculopathy	1	2.6
Anemia	1	2.6
OSAS	1	2.6
Duodenal ulcer	1	2.6
Sjogren syndrome	1	2.6

*N* number of patients, *OSAS* obstructive sleep apnea syndrome. Comorbidities of 38 patients with fibromyalgia syndrome treated with medical cannabis at an Italian pain clinic

**Table 4:** Associated pain syndromes in 38 Italian patients with fibromyalgia syndrome treated with medical cannabis

Pain syndrome	***N*** = 38	
	Patients	%
Low back pain	9	23.7
Cervical pain	8	21.1
Headache	8	21.1
Hip pain	4	10.5
Knee pain	3	7.9
Pelvic pain	2	5.3
Coccyx pain	1	2.6
FBSS	1	2.6
Shoulder pain	1	2.6
Hand pain	1	2.6
Burning mouth syndrome	1	2.6

*FBSS* failed back surgery syndrome

### Dropouts

**Fig 1:**
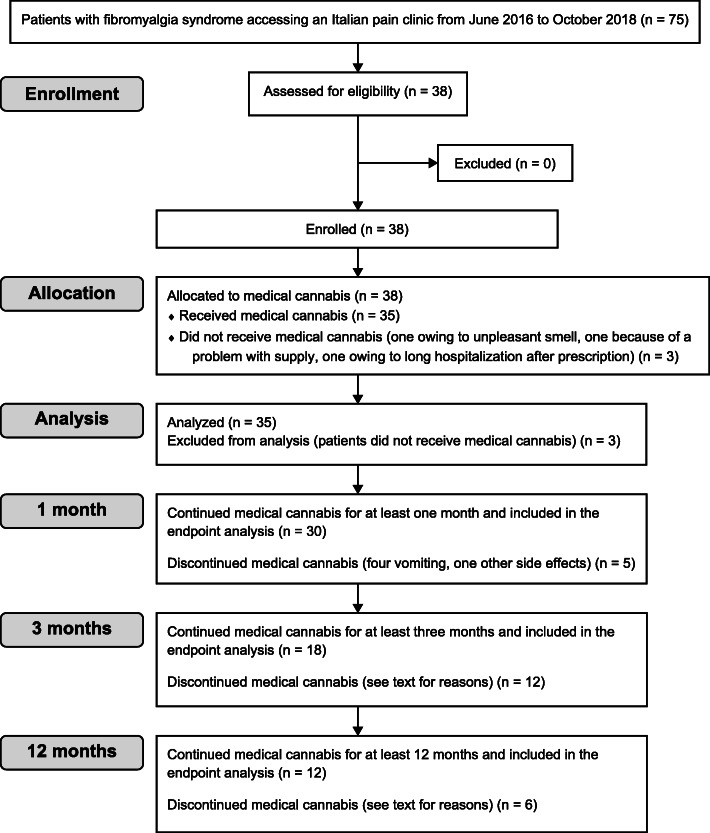
Consort flow chart diagram

**Fig. 2:**
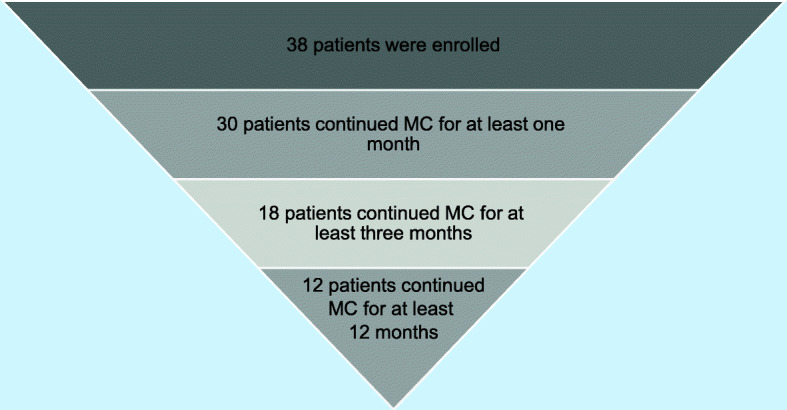
Number of patients enrolled and assessed at 1, 3, and 12 months. The number of patients affected by fibromyalgia syndrome continuing MC treatment for 1, 3, and 12 months in an Italian pain clinic. MC medical cannabis

Three patients did not begin MC therapy owing to its unpleasant scent, difficulty faced in an attempt to retrieve cannabis by the hospital pharmacy, and a long period of hospitalization immediately following prescription; these patients were excluded from analysis. In total, 35 patients were included in the analysis. Four patients vomited after intake of one sachet of cannabis; thus, therapy was immediately halted for these patients (Bedrocan 100 mg, *n* = 2; FM2 100 mg, *n* = 2). One of the four patients who vomited had anorexia. Additionally, one patient experienced several side effects after one dose (headache, nausea, vertigo, irritation, anger, increased sexual drive, insomnia, dry mouth, palpitations, abdominal distension, and erect nipples); thus, MC therapy was discontinued immediately. Thirty patients continued MC therapy for at least 1 month and were included in the endpoint analysis, 20 patients experienced analgesic effects, 17 patients experienced analgesic effects without side effects, three patients showed mild side effects and thus continued therapy until the first follow-up after 1 month but did not want to continue therapy afterwards owing to side effects; nine patients did not experience analgesic effects and had no, or mild, side effects and discontinued the MC treatment owing to ineffectiveness; one patient did not experience any analgesic effects but showed an improvement in sleep duration and continued therapy. Seventeen patients (48.6%) interrupted therapy owing to side effects. Eighteen patients continued therapy for at least 3 months, whereas 12 patients continued therapy for at least 12 months. After 3 months and before 12 months, six patients discontinued MC therapy. Among these patients, one could no longer come to the hospital for personal reasons, one moved to another region for family reasons, one interrupted MC therapy for work reasons, one could no longer obtain MC as the hospital pharmacy of her town no longer supplied MC therapy, one patient was worried about driving, and one patient interrupted therapy because she moved to another region for hospitalization for surgery (Fig. 1). Figure 2 summarizes the number of patients at various assessment times.

All patients began cannabis therapy in the form of decoction in accordance with Italian law. After titration, six responsive patients switched from decoction to vaporization (*n* = 2) or oil extract (*n* = 4), primarily due to the unpleasant taste of cannabis and disgust.

Table 5 shows the different MC cultivars used as starting therapy and summarizes the clinical treatment course. Twelve patients started with Bedrocan; five stopped therapy, while none of the responsive patients who began treatment with Bedrocan changed the therapy for clinical reasons. Eight patients started with Bediol; three stopped therapy, two switched to Bedrocan (for more efficacy on pain), and three switched to FM2 after Bediol became unavailable in the hospital pharmacy). Fifteen patients started therapy with FM2; six stopped therapy, and one switched to Bedrocan to achieve increased efficacy with regard to pain reduction. Three patients started therapy with Pedanios; one switched to FM2 to reduce AEs, and one switched owing to unavailability of Pedanios.

**Table 5:** Types of medical cannabis cultivars first prescribed to 38 Italian patients with fibromyalgia syndrome

MC cultivar starting therapy	Number of patients who started therapy	Number of patients who stopped therapy	Number of patients who switched therapy
Total number of patients	38	14	8
Bedrocan	12	5 (1 did not start therapy)	0
Bediol	8	3 (1 did not start therapy)	5 (2 to Bedrocan, 3 to FM2)
FM2	15	6 (1 did not start therapy)	1 to Bedrocan
Pedanios	3	0	2 to FM2

*MC* medical cannabis. The table shows the types of medical cannabis cultivars prescribed to 38 patients with fibromyalgia syndrome at an Italian pain clinic. The second column shows how many patients stopped therapy after the prescription of the various type of MC. The third column shows how many patients switched the variety of MC during the study period

### Analgesic efficacy

**Fig. 3:**
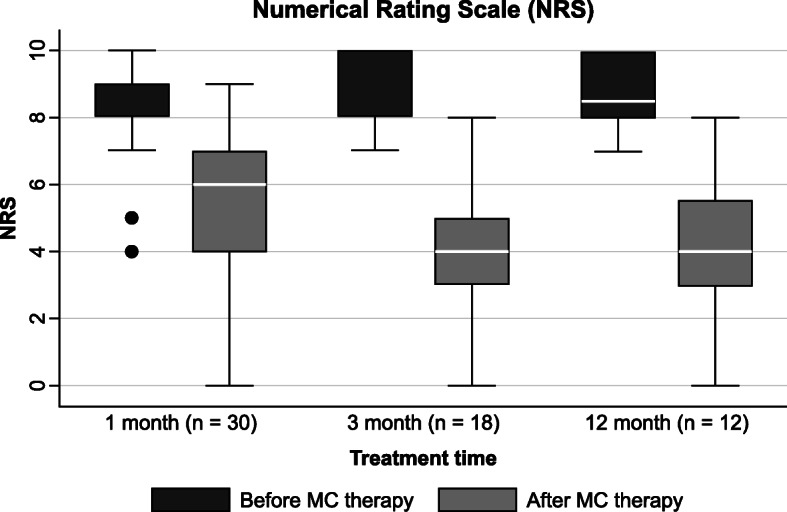
Numerical Rating Scale at 1, 3, and 12 months. NRS boxplots before and after MC therapy at 1, 3, and 12 months. Statistically significant improvements were registered for comparisons at 1, 3, and 12 months (*P <* 0.01). The boxes contain the middle 50% of the values; the thick white lines indicate the median; the whiskers connected by dashed lines to the boxes mark the minimum and maximum values except when there are outliners, which are specifically indicated by circles. NRS Numerical Rating Scale, MC medical cannabis

MC therapy significantly reduced pain intensity at 1, 3, and 12 months by at least 30% (Table 6). MC treatment was effective in 17 patients (Fig. 3). Five patients (14%) had a decrease in pain intensity of ≥ 30% or < 50%, whereas 12 patients (34%) had a decrease in pain intensity of ≥ 50%.

**Table 6:** The analgesic efficacy of medical cannabis therapy at 1, 3, and 12 months

	Prior MC	With MC	***P***	Prior MC	With MC	***P***	Prior MC	With MC	***P***
NRS	1 month, 30 patients			3 months, 18 patients			12 months, 12 patients		
Median	8	6	<0.01	8	4	<0.01	8.5	4	<0.01
IQR	1.25	3.25		2	2.25		2	2.75	
Mean	8	6		8	4		8.5	4	
± SD	1.4	2.3		1	1.8		1.2	2.1	

*MC* medical cannabis, *IQR* interquartile range, *±* plus/minus, *SD* standard deviation. The analgesic efficacy was measured with numerical rating scale (NRS) in 38 patients with fibromyalgia syndrome treated with medical cannabis at an Italian pain clinic. Wilcoxon signed-rank test was used to evaluate differences between time points

### Side effects

Side effects leading to an interruption of MC therapy occurred in 17 patients (48.6%); all side effects were quickly reversed after MC cessation (Table 7). The most common side effect was mental confusion; however, some patients reported more than one side effect. Patients who continued treatment experienced mild side effects, such as somnolence, which were not mentioned at the 3-month follow-up. No patients had serious side effects as per FDA definition.

**Table 7:** Side effects following medical cannabis therapy in a cohort of 35 Italian patients with fibromyalgia

Side effect	35 patients		18 patients		12 patients	
	***N***	%	***N***	%	***N***	%
Duration of therapy	After one day to 1 month		At 3 months		At 12 months	
Mental confusion	13	37				
Nausea/vomiting	5	14				
Vertigo/dizziness	5	14				
Restlessness/irritation	5	14				
Palpitations	4	11				
Somnolence	4	11				
Dry mouth	2	5				
Insomnia	2	5				
Nightmares	1	2				
Increased hunger	1	2				
Euphoria	1	2				
Headache	1	2	1	3%		
Increased sexual drive	1	2				
Abdominal distension	1	2				
Erect nipples	1	2				
Fatigue	1	2				
Anger	1	2				
Decreased creativity			1	3%		
Memory impairment					2	16%

*N* number of patients. Side effects after medical cannabis therapy in a cohort of 38 patients with fibromyalgia syndrome treated with medical cannabis at an Italian pain clinic: first column side effects experienced in 35 patients after 1-month medical cannabis therapy, second column side effects experienced in 30 patients after 3 months medical cannabis therapy, third column side effects experienced in 12 patients after 12 months medical cannabis therapy

### Effects on disability

**Fig. 4:**
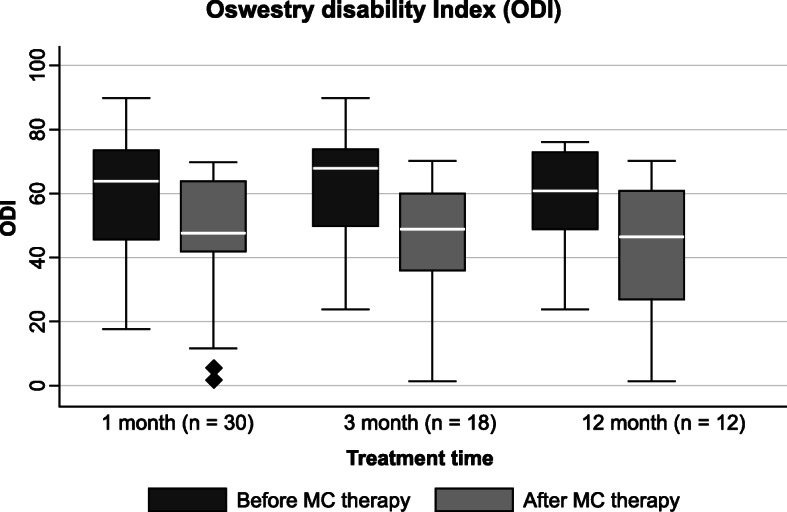
Oswestry Disability Index at 1, 3, and 12 months. ODI boxplots before and after MC therapy at 1, 3, and 12 months. Statistically significant improvements were registered for comparisons at 1, 3, and 12 months (*P <* 0.01). The boxes contain the middle 50% of the values; the thick white lines indicate the median; the whiskers connected by dashed lines to the boxes mark the minimum and maximum values except when there are outliners, which are specifically indicated by circles. ODI Oswestry Disability Index, MC medical cannabis

A significant improvement was observed in ODI at all time points (Table 8, Fig. 4).

**Table 8:** Medical cannabis effect on disability after 1, 3, and 12 months in Italian fibromyalgia patients

	Prior MC	With MC	***P***	Prior MC	With MC	***P***	Prior MC	With MC	***P***
ODI	1 month, *n* = 30			3 months, *n* = 18			12 months, *n* = 12		
Median	64%	48%	< 0.01	68%	49%	< 0.01	61%	47%	< 0.01
IQR	28	23.5		25	29.5		25	40	
Mean	64%	48%		68%	49%		61%	47%	
± SD	17.8	19.1		18.5	21.7		18.3	22.2	

*IQR* interquartile range, *±* plus/minus, *SD* standard deviation, *MC* medical cannabis, *ODI* Oswestry Disability Index. The disability was measured with Oswestry Disability Index (ODI) in a cohort of 38 patients with fibromyalgia syndrome treated with medical cannabis at an Italian pain clinic. Wilcoxon signed-rank test was used to evaluate differences between time points

### Effect on mood disorders

**Fig. 5:**
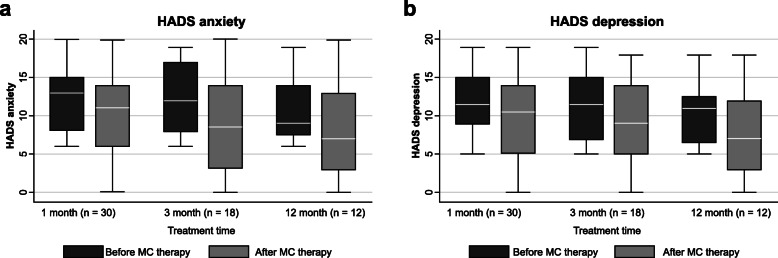
Hospital Anxiety and Depression Scale at 1, 3, and 12 months. **a** HADS anxiety and **b** HADS depression boxplots before and after MC therapy at 1, 3, and 12 months. No statistically significant improvements were registered for comparisons at 1, 3, and 12 months *(P <* 0.01). The boxes contain the middle 50% of the values; the thick white lines indicate the median; the whiskers connected by dashed lines to the boxes mark the minimum and maximum values except when there are outliners, which are specifically indicated by circles. HADS hospital anxiety and depression scale, MC medical cannabis

No significant improvements were observed in HADS anxiety indexes at all time points (1 month, *P* = 0.023; 3 months, *P* = 0.027). No significant improvements were observed for HADS depression indexes at all time points (1 month, *P* = 0.020; 3 months, *P* = 0.027; Table 9, Figure 5).

**Table 9:** Medical cannabis effects on mood disorders at 1, 3, and 12 months in Italian fibromyalgia patients

	Prior MC	With MC	***P***	Prior MC	With MC	***P***	Prior MC	With MC	***P***
	1 month, 30 patients			3 months, 18 patients			12 months, 12 patients		
HADS anxiety									
Median	13	11	>0.01	12	8.5	>0.01	9	7	>0.01
IQR	7.5	8.25		9.25	11.5		7.25	10.5	
Mean	13	11		12	8.5		9	7	
± SD	4.3	5.6		4.6	6.3		4.7	6.3	
HADS depression									
Median	11.5	10.5	>0.01	11,5	9	>0.01	11	7	>0.01
IQR	6.75	9		8.25	10		6.5	10.5	
Mean	11.5	10.5		9	8.4		11	7	
± SD	4.2	5.8		4.3	5.9		3.9	5.7	

*MC* medical cannabis, *HADS* Hospital Anxiety Depression Scale, *IQR* interquartile range, *±* plus/minus, *SD* standard deviation. Mood disorders were measured with Hospital Anxiety and Depression Scale (HADS) in a cohort of 38 patients with fibromyalgia syndrome treated with medical cannabis at an Italian pain clinic. Wilcoxon signed-rank test was used to evaluate differences between time points

### Effects on WPI and SyS

**Fig. 6:**
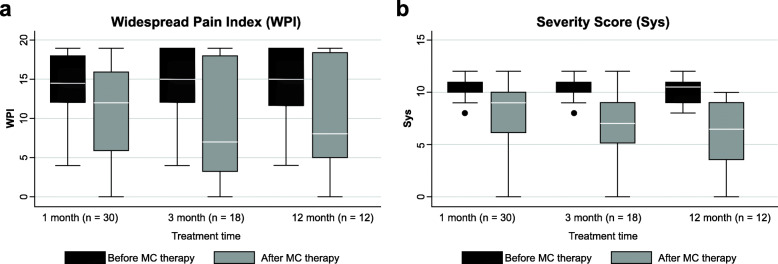
Widespread Pain Index and Severity Score at 1, 3, and 12 months. Boxplots of **a** WPI and **b** SyS before and during MC therapy at 1, 3, and 12 months. Statistically significant improvements in WPI were registered for comparisons at 1 and 3 months, but not at 12 months (*P* < 0.01). Statistically significant improvements of SyS were registered for comparisons at 1, 3, and 12 months (*P <* 0.01). The boxes contain the middle 50% of the values; the thick white lines indicate the median; the whiskers connected by dashed lines to the boxes mark the minimum and maximum values except when there are outliners, which are specifically indicated by circles. WPI widespread pain index, SyS severity score, MC medical cannabis

SyS improved significantly at all time points (Table 10). Additionally, WPI was significantly improved at 1 and 3 months after MC therapy, whereas no significant improvement was observed at 12 months (Fig. 6).

**Table 10:** Effects of medical cannabis on fibromyalgia syndrome at 1, 3, and 12 months

	Prior MC	With MC	***P***	Prior MC	With MC	***P***	Prior MC	With MC	***P***
	1 month, 30 patients			3 months, 18 patients			12 months, 12 patients		
Widespread pain index (WPI)									
Median	14.5	12	<0.01	15	7	<0.01	15	8	>0.01
IQR	6.25	10.5		7.25	15.5		7.75	14.25	
Mean	14.4	10.9		15	7		15	8	
± SD	3.9	6.3		4.2	7.1		4.8	7	
Severity score (SyS)									
Median	11	9	<0.01	11	7	<0.01	10.5	6.5	<0.01
IQR	1.25	4		1.5	4		2	6.25	
Mean	11	9		11	7		10.5	6.5	
± SD	1.3	2.9		1.3	3.2		1.3	3.3	

*MC* medical cannabis, *IQR* interquartile range, *±* plus/minus, *SD* standard deviation. Fibromyalgia syndrome was measured with Widespread Pain Index (WPI) and with Severity Score (SyS). Wilcoxon signed-rank test was used to evaluate differences between time points

### MC dosage

The daily dosages of milled MC used for treatment were as follows:

- THC-dominant MC therapy (Bedrocan, FM1, and Pedanios): median 200 mg (IQR 200), mean 240 mg (SD ± 144) per day in 1–3 administrations (minimum daily dosage: 50 mg; maximum daily dosage: 600 mg)
- Hybrid MC therapy (Bediol and FM2): median 400 mg (IQR 200), mean 383 mg (SD ± 162) per day in 1–3 administrations (minimum daily dosage: 100 mg; maximum daily dosage: 600 mg)

Tables 11 and 12 show the dosages of MC administered to the 18 patients who continued therapy for a minimum of 3 months.

**Table 11:** Dosage of THC and CBD administered with dominant THC medical cannabis to fibromyalgia patients

Patient	THC-dominant MC (Bedrocan, FM1, Pedanios), ***N***= 18			
	Decoction		Oil extract	
	THC (mg)	CBD (mg)	THC (mg)	CBD (mg)
Patient #1	22	0		
Patient #2	132	0		
Patient #3	22	0		
Patient #4			9.69	0
Patient #5	66	0		
Patient #6	100.8	2.4		
Patient #7				
Patient #8	53.4	1.2		
Patient #9	22	0		
Patient #10				
Patient #11	99	0	34.2	0
Patient #12	52.8	0.3		
Patient #13	46.2	0		
Patient #14	44	0	8.55	0
Patient #15	22	0		
Patient #16	35.2	0		
Patient #17				
Patient #18				
**Median**	**46.2**	**0**	**9.7**	**0**
**IQR**	**60.5**	**0.15**	**25.65**	**0**
**Mean**	**55.2**	**0.3**	**17.5**	**2**
**± SD**	**35.4**	**0.7**	**14.5**	**1.3**

*MC* medical cannabis, *THC* Δ-9-tetrahydrocannabinol, *CBD* cannabidiol, *N* number of patients, *IQR* interquartile range, *±* plus/minus, *SD* standard deviation. The table shows the quantity of Δ-9-tetrahydrocannabinol and cannabidiol administered with THC-dominant medical cannabis to 18 patients with fibromyalgia syndrome treated with medical cannabis for at least 3 months at an Italian pain clinic. THC dominant medical cannabis is the one with a dominant content of Δ-9-tetrahydrocannabinol

**Table 12:** Dosage of THC and CBD administered with hybrid medical cannabis to Italian fibromyalgia patients for at least 3 months

Patient	Hybrid MC (Bediol and FM2), ***N*** = 18			
	Decoction		Oil extract	
	THC (mg)	CBD (mg)	THC (mg)	CBD (mg)
Patient #1	6	8	0.66	0.55
Patient #2				
Patient #3	24	32		
Patient #4	36	48	3.9	3.3
Patient #5	23.6	38		
Patient #6			3.12	2.64
Patient #7	6	8	0.78	0.66
Patient #8	33	50		
Patient #9	26.4	40		
Patient #10	39.6	60		
Patient #11			4.68	3.96
Patient #12	23.6	38	1.56	1.32
Patient #13				
Patient #14				
Patient #15				
Patient #16	19.8	30		
Patient #17			1.8	1.65
Patient #18	19.8	30		
**Median**	**23.6**	**38**	**1.8**	**2**
**IQR**	**13.2**	**18**	**3.12**	**2.64**
**Mean**	**23.4**	**34.7**	**2.4**	**2**
**± SD**	**10.7**	**16.1**	**1.6**	**1.3**

*MC* medical cannabis, *THC* Δ-9-tetrahydrocannabinol, *CBD* cannabidiol, *N* number of patients, *IQR* interquartile range, *±* plus/minus, *SD* standard deviation. The table shows the quantity of THC and CBD administered with hybrid medical cannabis to 18 patients with fibromyalgia syndrome treated with medical cannabis for at least 3 months at an Italian pain clinic. Hybrid medical cannabis is the one with a similar content of Δ-9-tetrahydrocannabinol and cannabidiol

### Tolerance

Table 13 shows the dosage modification of MC for the 12 patients who continued MC treatment for 12 months. In four patients (33.3%), dosage reduction relative to that administered after 3 months of therapy was performed. However, due to the small sample size, a statistical analysis could not be performed. A reduction in the MC dosage was observed in three patients (25%). Additionally, one patient (8.3%) increased the MC dosage at 12 months, whereas eight patients (6.6%) maintained the same dosage at 12 months.

**Table 13:** Modification of MC dosage in 12 Italian fibromyalgia patients after 3 and 12 months of treatment

Patient: therapy regimen	***N*** = 12			
	After 3 months of MC therapy		After 12 months of MC therapy	
	THC (mg)	CBD (mg)	THC (mg)	CBD (mg)
Patient #2: Bedrocan decoction	132		66	
Patient #4: Bedrocan oil extract	9		9	
Patient #5: Bedrocan decoction	66		49.5	
Patient #6: FM1 vaporization	106.8		132	
Patient #8: FM1 decoction	53.4		50.4	
Patient #11: Bedrocan oil extract	31.2		31.2	
Patient #12: Pedanios decoction	52.8		52.8	
Patient #13: Bedrocan decoction	46.2		46.2	
Patient #14: Bedrocan oil extract	8.55		8.58	
Patient #15: FM1 decoction	27.6		16.8	
**Median**	**49.5**		**47.85**	
**IQR**	**53.25**		**41.25**	
**Mean**	**53.4**		**46.2**	
**± SD**	**40**		**36.1**	
Patient #16: Bediol vaporization	28.35	36	28.35	36
Patient #17: Bediol oil extract	1.95	1.95	2.1	1.8

*MC* medical cannabis, *THC* Δ-9-tetrahydrocannabinol, *CBD* cannabidiol, *N* number of patients, *IQR* interquartile range, *±* plus/minus, *SD* standard deviation. The table shows the quantity of THC and CBD used in a cohort of 12 patients with fibromyalgia syndrome treated with medical cannabis for at least 12 months at an Italian pain clinic. In the first column, you can see the quantity of MC used after a 3-month therapy, the column on the right shows the quantity of medical cannabis used by those same patients after 12 months MC treatment

### Drug use

Table 14 shows the number of patients taking different drugs at 3 and 12 months. Prior to MC therapy, all patients responsive to MC were taking one or more drugs. After 3 and 12 months of MC therapy, 33.3% and 66.7% of patients were taking MC alone, respectively. After 12 months of MC therapy, one patient, who experienced no analgesic effect, but had an increase in sleep hours, was taking four drugs. Two patients continued taking duloxetine for depression, and one patient continued to take low-dose steroids for associated psoriatic arthritis.

**Table 14:** Number of Italian patients taking pharmacological treatment prior to, and during, medical cannabis therapy

	***N*** = 18		***N*** = 12	
	Prior to MC therapy	At a 3-month MC therapy	At a 3-month MC therapy	At a 12-month MC therapy
**Analgesic medication**				
Acetaminophen (ACE)	4 (22.2%)	1 (5.5%)	1 (8.3%)	0
NSAIDs	3 (16.6%)	2 (11.1%)	1 (8.3%)	0
Steroids	1 (5.5%)	1 (5.5%)	1 (8.3%)	1 (8.3%)
Weak opioids	2 (11.1%)	2 (11.1%)	2 (16.6%)	0
Strong opioids	5 (27.7%)	3 (16.6%)	3 (25%)	0
**Drug**				
Amitriptyline	0	0	0	0
Gabapentin	0	0	0	0
Pregabalin	6 (33.3%)	3 (16.6%)	2 (16.6%)	1 (8.3%)
Duloxetine	7 (38.8%)	6 (33.3%)	5 (41.6)	3 (25%)
Venlafaxine	2 (11.1%)	1 (5.5%)	1 (8.3%)	0
Milnacipran	1 (5.5%)	1 (5.5%)	0	0
Benzodiazepines	7 (38.8%)	2 (11.1%)	2 (16.6%)	1 (8.3%)
Muscle relaxants	1 (5.5%)	0	0	0
**No drugs**	0	6 (33.3%)	6 (33.3%)	8 (66.7%)

*N* number of patients, *MC* medical cannabis, *NSAIDs* nonsteroidal anti-inflammatory drugs. The table shows the number of patients with fibromyalgia syndrome taking analgesic medications and drugs before starting medical cannabis treatment and during medical cannabis treatment at an Italian pain clinic

### Withdrawal

Symptoms of withdrawal were assessed in 30 patients who continued MC treatment for at least one month. All 30 patients interrupted their therapy for a period of time due to MC supply issues. Two patients (6%) appeared agitated and nervous after stopping MC, which may have represented symptoms of withdrawal.

## Discussion

In this study, the efficacy and AEs of MC in patients with FMS were evaluated. The results suggest that MC may have applications as an alternative treatment for FMS patients who are unresponsive to conventional therapy.

The findings of this study could be compared with the results from the few previous studies of MC in patients with FMS. Moreover, the demographic and clinical features of the 75 patients accessed in the pain clinic revealed that those included in the study were slightly older, and since they had received an FMS diagnosis two years prior to the MC therapy being available, it is likely that they had underwent all conventional treatments available in Italy at that time. The median duration of FMS since MC prescription was approximately 6.5 years, which was longer than that reported in two recent studies (5 and 4.2 years, respectively) (Fiz et al. 2011; Habib and Artul 2018). Additionally, the patients in this study were older (approximately 56 years) than those in other studies (Sagy et al. 2019; Fiz et al. 2011; Skrabek et al. 2008; Ware et al. 2010; Walitt et al. 2016; Habib and Artul 2018; Habib and Avisar 2018), and most patients in the current study were women, which was similar to results in other studies (Sagy et al. 2019; Fiz et al. 2011; Skrabek et al. 2008; Ware et al. 2010; Walitt et al. 2016; Habib and Artul 2018). Considering that the female-to-male ratio among patients with FMS is 3:1 (Queiroz 2013; Wolfe et al. 1995), the results of this study showed that most patients with FMS presenting to pain clinics are women. Notably, the percentage of patients reporting a headache was much lower than that in an Israeli study (Habib and Artul 2018) and in another study (Fiz et al. 2011). In contrast, the percentage of patients with irritable bowel syndrome in the current study was similar to that in the previous study (Habib and Artul 2018), whereas the median BMI of patients in this study was lower than that in other studies (Sagy et al. 2019; Walitt et al. 2016). Importantly, baseline NRS in this study was higher than the pain scores in other studies (Skrabek et al. 2008; Walitt et al. 2016), suggesting that the population in the current study had a greater pain intensity. Meanwhile, the assessment of pain, in a previous study (Habib and Artul 2018), using the Revised Fibromyalgia Impact Questionnaire (FIQR), may have caused differences between the studies. Moreover, median WPI and SyS in the current study were slightly higher than those in a controlled randomized study (Walitt et al. 2016). One strength of the current study was evaluation of the effects of long-term therapy (12 months) in a controlled hospital setting. In contrast, previous studies have evaluated the effects of MC at 6 months (Sagy et al. 2019), 2 h (Fiz et al. 2011), and 1–3 h (Walitt et al. 2016). Additionally, a randomized, double-blind, placebo-controlled trial on the use of nabilone for the treatment of pain in FMS evaluated the effects of the drug after 2 or 4 weeks of therapy (Skrabek et al. 2008). Other studies evaluated the effects of MC in patients who had already received therapy with MC with a mean treatment duration of approximately 10.4 months (Habib and Artul 2018).

One of the main findings of the current study was efficacy with regard to pain. Approximately half of the patients reported efficacy, with 34.2% opting to continue MC therapy for a long period (12 months). Most patients reporting response to therapy (~70%) showed decreased pain intensity by at least 50%. Moreover, long-term MC treatment was associated with interruption of conventional FMS drugs. Similar to a previous study (Romano and Hazekamp 2013), 66.7% of responsive patients used MC alone, whereas 50% of patients discontinued the use of other medications, and 46% reduced the dose/number of medications by at least 50%. Moreover, the findings in this study showed that NRS decreased by 5.1 points within three months and 4.3 points at 12 months. A reduction by 2 points was observed after 1-month MC therapy, a similar reduction (by 2.04 points) was observed in another study evaluating the treatment with nabilone for 4 weeks (Skrabek et al. 2008). In studies evaluating short-term therapy for FMS, a reduction in pain was also detected (Fiz et al. 2011), and only small analgesic responses were detected after a single inhalation. However, no differences were noted between nabilone and amitriptyline regarding pain (Ware et al. 2010). Reductions in pain have been reported in other studies of long-term MC treatment (Sagy et al. 2019; Habib and Artul 2018).

In this study, approximately half of patients experienced AEs, leading to interruption of MC treatment. However, few serious AEs were experienced following long-term therapy (12 months) in responsive patients, most of whom reported no or mild side effects, whereas patients who were not responsive to therapy reported side effects that were reversed immediately after treatment cessation. Importantly, no patients reported serious side effects according to the FDA definition. Mental confusion was the major side effect causing interruption of therapy. However, considering the significance of cognitive symptoms in FMS, this side effect was difficult to estimate. After the first titration period with cannabis, where 37% of patients reported mental confusion, this side effect gradually disappeared. In long-term therapy, no patients reported mental confusion or other side effects. After 3 months of therapy, one patient reported decreased creativity. A recent study showed that low-potency cannabis had no impact on creativity, whereas high-potency cannabis impaired divergent thinking (Kowal et al. 2015). In this study, only one patient had decreased creativity; therefore, it was not possible to draw conclusions regarding the potential effects of MC on creativity. Although two patients reported memory deterioration, whether this was dependent on FMS, other neurologic diseases, or cannabis use, was difficult to infer. A recent review showed that working memory was significantly impaired following acute exposure to cannabis. Additionally, this review showed that these deficits were resolved with sustained abstinence (Crean et al. 2011). In other studies, the most frequent reported AEs were dry mouth, red eyes, hunger, drowsiness, vertigo, ataxia, a drug-induced high, coughing, sore throat, bad taste, dyspnea, dizziness, headache, nausea, vomiting, somnolence, constipation, insomnia, sedation, tachycardia, and hypotension (Sagy et al. 2019; Fiz et al. 2011; Skrabek et al. 2008; Ware et al. 2010; van de Donk et al. 2019; Habib and Artul 2018). In the current study, similar to other studies, no patients reported serious side effects according to the FDA definition; only reversible side effects were observed.

Herein, disability was evaluated using ODI, and a statistically significant improvement was observed in MC responsive patients at all time points. The Revised Fibromyalgia Impact Questionnaire is a validated questionnaire that is commonly used to assess health-related quality-of-life in patients with FMS (Bennet et al. 2009). However, this questionnaire was not used here, instead, ODI, which is commonly applied to assess disability in patients with lower back pain, was employed. Since lower back pain is the most common pain syndrome treated in our pain clinic, daily clinical practice ensures every patient accessing the pain clinic completes an ODI questionnaire. Notably, many patients included in the study had lower back pain (23.7%), and all MC-treated patients had to complete this questionnaire at each monthly follow-up. Although ODI is not generally used to assess FMS patients, in a recent study evaluating the prevalence of the FMS phenotype in patients with spinal pain (Brummett et al. 2013), ODI was used to evaluate disability in a particular FMS population affected by lower back pain. However, considering that ODI has not been validated for FMS, the results should be interpreted carefully with regard to patient disability.

No improvements in anxiety or depression were observed in the current study. However, perceived relief of mood disorders and anxiety was noted in a cross-sectional survey of cannabis use in patients with FMS (Fiz et al. 2011). In another survey, 62% and 85% of patients showed improvements in anxiety and depression, respectively (Habib and Avisar 2018). Meanwhile, no differences were noted in mood disorders following treatment with nabilone and amitriptyline (Ware et al. 2010). In a trial on nabilone use in patients with FMS, a significant reduction in anxiety was observed by FIQR (Skrabek et al. 2008), and similar results were observed for MC in patients with FMS (Habib and Artul 2018).

In this study, a significant reduction in the WPI was observed at 1 and 3 months, but not at 12 months of MC therapy. Sys improved at all time points. No other studies evaluated WPI and Sys in patients with FMS receiving MC treatment.

Moreover, the median daily dose of consumed MC was lower than that described in other studies (Habib and Artul 2018; Habib and Avisar 2018). A prospective observational study of patients with FMS used an initiation dosage of 670 mg/day MC and 1000 mg/day MC at 6 months of follow-up; while the median THC and CBD content in the administered cultivar at 6 months was 140 and 39 mg/day, respectively (Sagy et al. 2019). In contrast, in the current study, the THC content in the administered THC-dominant cultivar was lower, and the content of THC + CBD in the administered hybrid MC cultivar was also lower in the decoction and oil extract. In an Israeli survey (Habib and Avisar 2018) with 383 participants, the mean dose of MC was 31.4 ± 16.3 g/month. Hence, considering that other studies examined the effects of unlicensed cannabis on patients with chronic pain, without any knowledge of the amount of consumed cannabis (Fiz et al. 2011), defining the median daily MC dosage may be quite valuable. Thus, another strength of this study was that the findings were based on the contents of THC and CBD. The findings obtained herein revealed that a smaller quantity of THC was required with the oil extract than with the decoction. However, because of the small sample size and potential bias, the results of this study should be interpreted with caution.

Importantly, no tolerance effect was observed in the current study. After the titration period, which lasted approximately 1–3 months, an increase in the dosage of therapy was not required, even after 12 months of therapy. Furthermore, in some cases (*n* = 4), a decrease in dosage was possible. This finding is important for long-term therapy as FMS can affect many young patients. Moreover, these results may suggest that patients with FMS could remain on relatively low doses of MC, in contrast to opiates. This finding is similar to that of another study, which showed that there were no tolerance effects in recreational cannabis users (Bedi et al. 2010). However, since the study settings were entirely different, direct comparisons cannot be made. Additionally, statistical significance could not be assessed due to the small sample size.

At the end of 2016, MC was unavailable in Italy for several weeks. As a result, the hospital pharmacy could not dispense the prescribed MC supply to patients. During this period, two patients appeared agitated and nervous, which could be symptoms of withdrawal (Bonnet and Preuss 2017). No other patients showed symptoms that could be attributed to withdrawal.

Certain limitations were noted in the current study. First, this study was retrospective in nature, hence, only previously collected data was available for evaluation, without the ability to evaluate disability with more appropriate questionnaires. However, the data was readily collected from self-administered questionnaires, the scores for which were calculated by a nurse, which were then recorded by the physician on the medical report during follow-up. Moreover, the retrospective nature of the study may have introduced patient selection bias. Second, potential bias may have occurred regarding patient inclusion in this study. Only patients with FMS who were resistant to conventional therapy were enrolled. Thus, further evaluation of MC treatment in all patients with FMS would prove meaningful. Third, the use of per protocol analysis was another limitation of the current study. Since the intent-to-treat analysis, in which all patient data are considered regardless of dropout, was not used during all observations, the efficacy of MC was limited to patients who continued the treatment, whereas patients who dropped out of the study were not considered. The per protocol analysis may reflect the maximum potential benefits of a treatment, which could overestimate MC therapy. Furthermore, the absence of a control group, the small sample size, use of different MC types, and routes of administration, also represent additional study limitations. Both control and placebo groups are required to determine the beneficial effects of cannabis in patients with FMS. However, inclusion of a placebo group may be challenging due to the typical aroma of herbal cannabis; thus, prior randomized controlled trials have been conducted with synthetic cannabis (Skrabek et al. 2008; Ware et al. 2010). In fact, a recently randomized placebo-controlled four-way crossover trial was conducted using a Bedrocan cannabis variety, which was used after selective removal of cannabinoids; however, the terpene profile, which is responsible for the smell and taste of cannabis (Walitt et al. 2016), was retained. The effects of cannabis were evaluated after selective removal was achieved. Another limitation of this study is that some patients were treated with MC for FMS but had coexisting pain syndromes. Thus, the treatment effects could have been related to chronic pain etiologies or comorbidities other than FMS. Additionally, although the questionnaire was not filled out at the physician’s office, but was self-administered, the results of the questionnaire were not kept confidential from the physician. Accordingly, patients could claim that improvement has occurred in front of a physician (observer-expectancy effect) or could claim improvement to continue obtaining the MC prescription. The choice to evaluate the efficacy of MC therapy in patients with FMS was related to the fact that treatment of FMS is very challenging with pharmacological conventional therapy often unsuccessful; therefore, it is important to share clinical experiences to alleviate pain and improve the quality-of-life of patients who have few therapeutic options. Collectively, the results of the current study could not be generalized to represent all patients with FMS, despite similarities between the findings of this study and those of others. Nonetheless, these results suggest that cannabinoids could be administered as a treatment agent for FMS. However, as recommended in a recent review (van de Donk et al. 2019), further clinical trials should be conducted.

## Conclusions

The current study revealed the positive effects of MC therapy in some patients with FMS and resistance to conventional treatment. Thus, cannabinoids may be considered for FMS treatment, although several side effects may still occur. Further studies are warranted to confirm these findings.

## Data Availability

The datasets used and/or analyzed during the current study are available from the corresponding author on reasonable request.

## Abbreviations

FMS: Fibromyalgia syndrome
ACR: American College of Rheumatology
WPI: Widespread Pain Index
SSS: Symptom Severity Score
THC: Δ-9-tetrahydrocannabinol
CBD: Cannabidiol
MC: Medical cannabis
NRS: Numerical Rating Scale
ODI: Oswestry Disability Index
PSD: Polysymptomatic Distress Scale
HADS: Hospital Anxiety and Depression Scale
Sys: Severity score
NHS: National Hospital Service
BMI: Body mass index
FIQ: Fibromyalgia Impact Questionnaire
FIQER: Revised Fibromyalgia Impact Questionnaire
IQR: Interquartile range

## References

[CR1] Andreae MH, Carter GM, Shaparin N, Suslov K, Ellis RJ, Ware MA, Abrams DI, Prasad H, Wilsey B, Indyk D, Johnson M, Sacks HS (2015). Inhaled cannabis for chronic neuropathic pain: a meta-analysis of individual patient data. J Pain.

[CR2] Arnold LM, Fan J, Iyengar SK (2013). The fibromyalgia family study: a genome-wide linkage scan study. Arthritis Rheumatol.

[CR3] Aviram J, Samuelly-Leichtag G (2017). Efficacy of cannabis-based medicines for pain Management: a systematic review and meta-analysis of randomized controlled trials. Pain Physician.

[CR4] Banerjee S, McCormack S (2019). Medical cannabis for the treatment of chronic pain: a review of clinical effectiveness and guidelines.

[CR5] Bedi G, Foltin RW, Gunderson EW (2010). Efficacy and tolerability of high-dose dronabinol maintenance in HIV-positive marijuana smokers: a controlled laboratory study. Psychopharmacology (Berl).

[CR6] Bellato E, Marini E, Castoldi F, Barbasetti N, Mattei L, Bonasia DE, Blonna D (2012). Fibromyalgia syndrome: etiology, pathogenesis, diagnosis and treatment. Pain Res Treat.

[CR7] Bennet RM, Friend R, Jones KD (2009). The Revised Fibromyalgia Impact Questionnaire (FIQR): validation and psychometric properties. Arthritis Res Ther.

[CR8] Bonnet U, Preuss UW (2017). The cannabis withdrawal syndrome: current insights. Subst Abuse Rehabil.

[CR9] Branco JC, Bannwarth B, Failde I, Carnbonell JA (2010). Prevalence of fibromyalgia: a survey in five European countries. Semin Arthritis Rheum.

[CR10] Brummett CM, Goesling J, Tsodikov A, Meraj TS, Wasserman RA, Clauw DJ, Hassett AL (2013). Prevalence of the fibromyalgia phenotype in spine pain patients presenting to a tertiary care pain clinic and the potential treatment implications. Arthritis Rheum..

[CR11] Costantini M, Musso M, Viterbori P, Bonci F, Del Mastro L, Garrone O, Venturini M, Morasso G (1999). Detecting psychological distress in cancer patients: validity of the Italian version of the Hospital Anxiety and Depression Scale. Support Care Cancer..

[CR12] Crean RD, Crane NA, Mason BJ (2011). An evidence based review of acute and long-term effects of cannabis use on executive cognitive functions. J Addict Med.

[CR13] Croft PR (2002). The epidemiology of chronic widespread pain. J Musculoskelet Pain.

[CR14] Decreto ministeriale 23 gennaio 2013. Aggiornamento delle tabelle contenenti l'indicazione delle sostanze stupefacenti e psicotrope, di cui al decreto del Presidente della Repubblica 9 ottobre 1990, n. 309 e successive modificazioni e integrazioni. Inserimento nella Tabella II, Sezione B, dei medicinali di origine vegetale a base di Cannabis (sostanze e preparazioni vegetali, inclusi estratti e tinture). [Italian law: Ministerial decree 23rd January 2013. Update of tables containing the indication of narcotics and psychotropic substances, from Republic President decree 9th of October 1990, n 309 and subsequent modifications and integrations. Inclusion in Table II, Section B, of Cannabis plant derived drugs (vegetal substances and preparations, including extracts and titures.]

[CR15] Deshpande A, Mailis-Gagnon A, Zoheiry N, Lakha SF (2015). Efficacy and adverse effects of medical marijuana for chronic noncancer pain: systematic review of randomized controlled trials. Can Fam Physician.

[CR16] ElSohly M, Gul W, Pertwee R (2014). Constituents of Cannabis sativa. Handbook of Cannabis.

[CR17] Fiz J, Duran M, Capella D, Carbonell J, Farré M (2011). Cannabis use in patients with fibromyalgia: effect on symptoms relief and health-related quality of life. Plos One.

[CR18] Gieringer D, St. Laurent J, Goodrich S (2008). Cannabis vaporizer combines efficient delivery of THC with effective suppression of pyrolytic compounds. J Cannabis Ther..

[CR19] Grotenhermen F, Russo E (2002). Cannabis and cannabinoids: pharmacology, toxicology and therapeutic potentials.

[CR20] Habib G, Artul S (2018). Medical Cannabis for the treatment of fibromyalgia. J Clin Rheumatol.

[CR21] Habib G, Avisar I (2018). The consumption of Cannabis by fibromyalgia patients in Israel. Pain Res Treat.

[CR22] Haefeli M, Elfering A (2006). Pain assessment. Eur Spine J.

[CR23] Haroutounian S, Ratz Y, Ginosar Y, Furmanov K, Saifi F, Meidan R, Davidson E (2016). The effect of medicinal cannabis on pain and quality-of-life outcomes in chronic pain: a prospective open-label study. Clin J Pain.

[CR24] Hazekamp A, Bastola K, Rashidi H, Bender J, Verpoorte R (2007). Cannabis tea revisited: a systematic evaluation of the cannabinoid composition of cannabis tea. J Ethnopharmacol..

[CR25] Ko GD, Bober SL, Mindra S, Moreau JM (2016). Medical cannabis–the Canadian perspective. J Pain Res.

[CR26] Kowal MA, Hazekamp A, Colzato LS, van Steenbergen H, van der Wee NJA, Durieux J, Manai M, Hommel B (2015). Cannabis and creativity: highly potent cannabis impairs divergent thinking in regular cannabis users. Psychopharmacology.

[CR27] Lunn M, Hughes RA, Wiffen PJ (2014). Duloxetine for treating painful neuropathy, chronic pain or fibromyalgia.

[CR28] Macfarlane GJ, Kronisch C, Dean LE, Atzeni F, Hauser W, Fluss E, Choi E, Kosek E, Amris K, Branco J, Dincer F, Leino-Arjas P, Lonley K, McCarthy M, Makri S, Perrot S, Sarzi-Puttini P, Taylor A, Jones GT (2016). EULAR revised recommendations for the management of fibromyalgia. Ann Rheum Dis.

[CR29] Mc Beth J, Macfarlane GJ, Hunt IM, Silman AJ (2001). Risk factors for persistent chronic widespread pain: a community-based study. Rheumatology.

[CR30] Mechoulam R, Mc Callum NK, Burstein S (1976). Recent advances in the chemistry and biochemistry of Cannabis. Chem Rev.

[CR31] Monticone M, Baiardi P, Ferrari S, Foti C (2009). Development of the Italian version of the Oswestry disability index (ODI-I): a cross-cultural adaptation, reliability and validity study. Spine.

[CR32] Mücke M, Phillips T, Radbruch L, Petzke F, Häuser W (2018). Cannabis-based medicines for chronic neuropathic pain in adults. Cochrane Database Syst Rev..

[CR33] National Pain Report (2014) Marijuana rated most effective for treating fibromyalgia. National Pain Report. http://nationalpainreport.com/marijuana-rated-most-effective-for-treating-fibromyalgia-8823638.html. Accessed 15 Jan 2020.

[CR34] Piper BJ, Beals ML, Abess AT, Nichols D, Martin M, Cobb CM, DeKeuster RM (2017). Chronic pain patients’ perspectives of medical cannabis. Pain.

[CR35] Queiroz L (2013). Worldwide epidemiology of fibromyalgia. Curr Pain Headache Rep.

[CR36] REGIONE PIEMONTE BU8S1 25/02/2016. Deliberazione della Giunta Regionale 15 febbraio 2016, n. 24-2920 Indirizzi procedurali ed organizzativi per l'attuazione della Legge Regionale n. 11 del 15 giugno 2015 - Uso terapeutico della canapa. [Regional law: Piedmont Region BU8S1 25/02/2016. Resolution of regional government 15 February 2016, n 24-2920. Procedural and organizational guidelines for the implementation of the regional law n. 11 15th June 2015 – Therapeutic use of hemp.]

[CR37] Romano L, Hazekamp A (2013). Cannabis oil: evaluation of an upcoming cannabis-based medicine. Cannabinoids.

[CR38] Sagy I, Bar-Lev L, Abu-Shakra M, Novack V (2019). Safety and efficacy of medical cannabis in fibromyalgia. J Clin Med.

[CR39] Schmidt-Wilcke T, Clauw DJ (2011). Fibromyalgia: from pathophysiology to therapy. Nat Rev Rheumatol.

[CR40] Skrabek RQ, Galimova I, Ethansand Dary K (2008). Nabilone for the treatment of pain in fibromyalgia. J Pain.

[CR41] Troutt WD, DiDonato MD (2015). Medical cannabis in Arizona: patient characteristics, perceptions and impressions of medical cannabis legalization. J Psychoactive Drugs.

[CR42] Üçeyler N, Sommer C, Walitt B, Häuser W (2013). Anticonvulsants for fibromyalgia.

[CR43] van de Donk T, Niesters M, Kowal MA, Olofsen E, Dahan A, van Velzen M (2019). An experimental randomized study on the analgesic effects of pharmaceutical-grade cannabis in chronic pain patients with fibromyalgia. Pain.

[CR44] Vincent A, Lahr BD, Wolfe F, Clauw DJ, Whipple MO, Oh TH, Barton DL, Sauver JS (2013). Prevalence of fibromyalgia: a population-based study in Olmsted County, Minnesota, utilizing the Rochester epidemiology project. Arthritis Care Res.

[CR45] Walitt B, Klose P, Fitzcharles MA, Phillips T, Hauser W (2016). Cannabinoids for fibromyalgia. Cochrane Database Syst Rev..

[CR46] Ware MA, Fitzcharles MA, Joseph I, Shir Y (2010). The effect of nabilone on sleep in fibromyalgia: results of a randomized controlled trial. Anaesth Analg.

[CR47] Wolfe F, Brahler E, Hinz A, Hauser W (2013). Fibromyalgia prevalence, somatic symptom reporting, and the dimensionality of polysymptomatic distress: results from a survey of the general population. Arthritis Care Res.

[CR48] Wolfe F, Brian BD, Walitt MD, Hauser W (2015). The use of polysymptomatic distress categories in the evaluation of fibromyalgia (FM) and FM severity. J Rheumatol.

[CR49] Wolfe F, Clauw DJ, Fitzcharles MA, Goldenberg DL, Hauser W, Katz RL, Mease P, Russell AS, Russell IJ, Walitt B (2016). Revision to the 2010/2011 fibromyalgia diagnostic criteria. Semin Arthritis Rheumatol.

[CR50] Wolfe F, Clauw DJ, Fitzcharles MA, Goldenberg DL, Katz RS, Mease P, Russell AS, Russell IJ, Winfield JB, Yunus MB (2010). The American College of Rheumatology preliminary diagnostic criteria for fibromyalgia and measurement of symptom severity. Arthritis Rheumatol.

[CR51] Wolfe F, Clauw DJ, Fitzcharles MA, Goldenberg DL, Katz RS, Mease P, Russell AS, Russell IJ, Winfield JB, Yunus MB (2011). Fibromyalgia criteria and severity scales for clinical and epidemiological studies: a modification of the ACR preliminary Diagnostic Criteria for Fibromyalgia. J Rheumatol.

[CR52] Wolfe F, Ross K, Anderson J, Russell IJ, Herbert L (1995). The prevalence and characteristics of fibromyalgia in the general population. Arthritis Rheumatol.

[CR53] Wolfe F, Walitt MD, Rasker J, Hauser W (2018). Primary and secondary fibromyalgia are the same: the universality of polysymptomatic distress. J Rheumatol.

[CR54] Yaseen K, Kaouk S, Bork D, Wilke WS, Gota C (2017). Polysymptomatic distress scale, widespread pain index, and symptoms severity scale, and their correlates in 169 patients with fibromyalgia syndrome. Ann Rheum Dis.

